# Progress in high-throughput screening for drug and material discovery in orthopedic diseases: a literature review

**DOI:** 10.7717/peerj.21587

**Published:** 2026-08-12

**Authors:** Aiwen Zhang, Qiang Wang, Wuxun Peng, Jian Zhang

**Affiliations:** 1 Emergency Department, The Affiliated Hospital of Guizhou Medical University, Guiyang, Guizhou, China; 2 Clinical Medical College, Guizhou Medical University, Guiyang, China

**Keywords:** High-throughput screening (HTS) technology, Orthopedic diseases, Drug discovery, Bone microenvironment simulation, Clinical translation

## Abstract

Orthopedic diseases are referred to as a series of conditions affecting the normal structure and function of the skeletal system. With a wide variety of types and a year-by-year increasing incidence, they have a severe impact on patients’ quality of life. Traditional research has made relatively significant progress in understanding the pathogenesis and treating orthopedic diseases, however, it remains constrained by limitations such as low-throughput experimentation, and difficulty in mimicking the complex bone microenvironment. In recent years, high-throughput screening (HTS) technology has been combined with experimental techniques and models (such as microfluidics, omics technologies, 3D bioprinting, artificial intelligence, and organoid models), which can be used to better simulate the real bone microenvironment and predict molecular activity. This combined approach has not only deepened our understanding of the mechanisms of orthopedic diseases, but also broken through the limitations of traditional drug development, which further accelerates drug discovery and clinical translation. This review aims to systematically summarize the research progress and applications of HTS in typical orthopedic diseases, including osteoporosis, osteoarthritis, bone defects, and bone tumors, to promote the development and clinical translation of novel therapeutic strategies for orthopedic disorders, and to provide references and guidance for researchers in orthopedic basic research, drug development, regenerative medicine, and clinical orthopedic practice. Accordingly, this review is intended for orthopedic researchers, clinicians and biomaterial scientists focusing on high-throughput screening and orthopedic therapeutic development.

## Introduction

In the 1980s, high-throughput screening (HTS) emerged as a technology capable of screening target molecules in a rapid and massive way. Due to its automation and high efficiency, HTS technology is commonly used to screen lead compounds (Wildey et al., 2017). Its principle involves using automated and miniaturized approaches to rapidly test and analyze the specific biological functions of samples (Shinn et al., 2019b). Compared with traditional screening methods, HTS technology offers advantages such as automation, low cost, high efficiency, low error rate, and high reproducibility (Mayr & Bojanic, 2009). Recently, HTS technology has gradually expanded its application into fields such as materials science and regenerative medicine (Li et al., 2025a; Lipreri et al., 2023), providing novel perspectives for mechanism exploration, drug development and clinical management of human orthopedic disorders (Shi et al., 2023). Modern HTS increasingly relies on the integration of microfluidics, 3D organoids and artificial intelligence to improve screening accuracy and efficiency (Shi et al., 2023; Li et al., 2026).

Orthopedic diseases can be broadly classified into three categories. The first category consists of diseases primarily characterized by bone metabolic disorders, such as osteoporosis and osteoarthritis (Shoback, 2007). Metabolic diseases mainly arise from the homeostatic imbalance between osteoblasts and osteoclasts, or from the disordered anabolic and catabolic metabolism of chondrocytes in conditions like osteoarthritis (OA) (Ebeling et al., 2022; Qin et al., 2025). The second category includes diseases marked by decreased bone regenerative capacity, such as bone defects and non-union. Abnormal bone regeneration is caused by reduced osteogenic differentiation ability of bone marrow mesenchymal stem cells (BMSCs) or osteoblasts (OB) (Salhotra et al., 2020). The third category comprises other diseases like bone tumors, which are induced by changes in the cellular microenvironment (Batoon & McCauley, 2021). In recent years, significant advancements have been made in research on bone metabolism, bone regeneration mechanisms, as well as cellular microenvironments, providing crucial theoretical and practical foundations for the clinical treatment of metabolic bone diseases, small bone defects, non-union fractures, and bone tumors.

Nevertheless, current clinical regimens for orthopedic disorders face considerable challenges (Gao et al., 2025; Chae & Cho, 2023). Conventional drugs often cause systemic side effects, poor targeting, and drug resistance, compromising their long-term efficacy. Additionally, traditional 2D cultures and simplified animal models fail to fully recapitulate the complex extracellular matrix, mechanical microenvironment, and spatial structure of native bone, resulting in poor preclinical predictivity and translational efficiency (Chae & Cho, 2023; Fischetti et al., 2026). While 3D bioprinting enables the construction of biomimetic bone models that better simulate *in vivo* physiological conditions (Hagenbuchner, Nothdurfter & Ausserlechner, 2021), such models remain limited by low-throughput workflows and inefficient screening (Shrestha et al., 2021). In orthopedic applications, 3D bioprinting has shown significant value in fabricating patient-specific scaffolds and tissue constructs (Freeman, Burdis & Kelly, 2021; Zheng et al., 2025). For example, in the field of large segmental bone defects, 3D-printed bioactive ceramic scaffolds with precisely controllable porosity have been proven to promote osseointegration and restore mechanical function in preclinical models (Zeng et al., 2025; Shen et al., 2022). In osteoarthritis research, layered 3D bioprinted osteochondral constructs and cartilage organoids have been developed to mimic the native cartilage microenvironment, providing a more physiologically relevant platform for drug screening and disease modeling (Gomes et al., 2024; Liang et al., 2022). In osteochondral regeneration, biphasic bioprinted scaffolds incorporating both chondrogenic and osteogenic cell populations have been confirmed to facilitate simultaneous repair of articular cartilage and subchondral bone defects (Zhang et al., 2024). Given these advantages and application scenarios, the integration of 3D bioprinted models with HTS platforms is critically essential to achieve large-scale drug screening under pathologically relevant conditions (Mi et al., 2023; Xu et al., 2025).

Recently, with the development of HTS technology, significant progress has been made in the prevention and treatment of orthopedic diseases. Mechanistically, combined with omics technologies, and other methods, HTS can rapidly sequence and identify biological markers such as DNA, RNA, and proteins, so as to better identify therapeutic targets for diseases (Batoon & McCauley, 2021; Parola, Neumeier & Reddy, 2018; Brommage et al., 2014; Topol, 2024). In the field of drug development, combined with corresponding platforms and models, HTS technology enables the identification, validation, and optimization of large numbers of compounds with similar bioactive functions to screen the most promising candidates (Hughes et al., 2011). To date, HTS technology has been widely applied in orthopedic diseases, however, its underlying principles, system construction, screening methods, and specific contributions to orthopedic diseases have not been fully elaborated. Therefore, this review systematically summarizes the technical characteristics and research progress of HTS, and highlights its application value and research prospects in various orthopedic diseases, aiming to offer theoretical support for the development of novel therapeutic strategies and clinical translational research in orthopedics.

### Survey methodology (PRISMA-aligned)

This work was conducted as a narrative literature review, structured in accordance with the PRISMA 2020 guidelines to ensure transparency and comprehensiveness in evidence synthesis.

### Data sources and search strategy

A systematic literature search was performed across the following electronic databases: PubMed, Web of Science, Embase, and Google Scholar (for supplementary gray literature). The search strategy combined keywords related to high-throughput screening and orthopedic conditions: “high-throughput screening” OR “HTS” AND “orthopedic” OR “orthopaedic” OR “osteoporosis” OR “bone defect” OR “bone tumor” OR “bone regeneration”. The publication time range was restricted to January 2000 to December 2025.

### Eligibility criteria

Studies were included based on the following criteria: Original research, communications, and reviews focusing on HTS applications in orthopedic diseases. Studies involving compound screening, biomaterial screening, target identification, or drug discovery for orthopedic conditions. Studies with clearly reported screening models, methods, or outcome measures.

Studies were excluded if they met any of the following criteria: Non-English publications. Basic cell or molecular studies without reported HTS applications. Duplicate publications, conference abstracts, or studies unrelated to the scope of this review.

## Results

### High-throughput screening

By relying on various experimental models and methodologies, HTS is an experimental system that integrates automated equipment and high-sensitivity detection technologies for data acquisition, analysis, and storage, and can simultaneously process thousands to millions of samples within a short period to screen for target molecules (Pereira & Williams, 2007).

The construction of a HTS technology platform relies on multiple core components such as the sample library, the experimental system, experimental methods and detection technologies, as well as the automated equipment. The sample library serves as the cornerstone of HTS technology and is indispensable for its application. HTS technology platforms feature a wide variety of sample libraries. Among them, the more widely used ones include approved drug libraries, synthetic compound libraries, natural compound libraries, material libraries, protein libraries, gene target libraries, *etc*. Selecting or constructing a suitable sample library can not only reduce screening costs but also improve screening efficiency (Paricharak et al., 2018; Wigglesworth et al., 2015; Kogej et al., 2013; Seo et al., 2018; Peña-García et al., 2016). The experimental system acts as the carrier for samples and reactions. The establishment of an experimental system determines the scale and efficiency of experiments. Significant progress has been made in platform construction, and numerous different experimental systems. Based on the screening targets, there have been various systems (organoid systems, and fluorescent cell 3D culture systems) and models (*Caenorhabditis elegans* disease models, and zebrafish disease models) (Ben-Yakar, 2019; Delvecchio, Tiefenbach & Krause, 2011; Wei et al., 2024). The experimental methods and detection technologies include the CCK-8 assay, surface plasmon resonance, mass spectrometry, high-throughput sequencing, fluorescence resonance energy transfer, enzyme-linked immunosorbent assay, functional whole-cell assays, and high-content screening. The development of these methods and technologies determines the accuracy and reliability of experiments (Entzeroth, Flotow & Condron, 2009; Alma’abadi et al., 2022). The automated equipment (including equipment for maintaining experimental operations and devices for data collection, analysis, and storage, such as fully automated robotic systems, data acquisition equipment, and data analysis software) has a significant impact on experimental costs and reproducibility (Shinn et al., 2019a; Dörr et al., 2016). These components collaborate to achieve reliable, efficient, accurate, and low-cost screening goals.

Currently, the most advanced HTS has been integrated with AI-driven automated platforms, 3D bioprinted microtissue models, as well as bionic bone micro-environment systems based on the organ chip (Paek et al., 2023; Lipreri et al., 2023; Wang & Jeon, 2022). It has gradually shifted from single-cell to multi-cell systems, from 2D to 3D technology levels, and from molecular-level studies to *in vivo* organisms. Multi-cell, 3D-level, and *in vivo* models can be applied to better simulate physiological or pathological states, leading to higher clinical translatability of screened target molecules (Juraski et al., 2023). Therefore, HTS technology has become one of the core technologies in modern medical advancements.

### Applications of HTS in orthopedic diseases

As an efficient and large-scale screening method, HTS technology can be used to rapidly and accurately identify target proteins, genes, signaling pathways, and small-molecule compounds. Orthopedic diseases (represented by osteoporosis, osteoarthritis, bone defects, and bone tumors) not only significantly affect patients’ motor function and quality of life, but also pose life-threatening risks in some progressive cases. The integration of HTS with advanced technologies (*e.g*., reporter gene systems, near-infrared fluorescent probes, molecular docking, and bioinformatics analysis) has greatly enhanced the efficiency and accuracy of target discovery and lead compound screening in orthopedic disease research, breaking through the limitations of traditional research methods. This section comprehensively reviews the application of HTS technology in the treatment of the above-mentioned orthopedic diseases, with a focus on the integration mode of HTS and high-end technologies, and conducts critical comparative analysis of the screened compounds to provide insights for future research and clinical transformation.

#### Osteoporosis

Osteoporosis (OP), a systemic bone metabolic disorder, can lead to the increased bone fragility and fracture risk, ranking as one of the global public health issues (Iñiguez-Ariza & Clarke, 2015). Traditional treatments primarily rely on oral medications such as bisphosphonates and teriparatide to control its progression (Ebeling et al., 2022). Current first-line therapies also include romosozumab and denosumab (Moshi et al., 2023). However, long-term use of bisphosphonates can lead to higher risks of atypical femoral fractures and drug-related osteonecrosis of the jaw (Gehrke et al., 2023). Oral administration of romosozumab may increase the risk of myocardial infarction, stroke, and cardiovascular death, particularly in patients with a history of cardiovascular disease (Lim, 2022). As a result, it has become a research priority to develop safer and more effective drugs or clinical lead compounds for preventing, diagnosing, and treating osteoporosis. The following sections detail the screening process, mechanism of action of typical OP-related compounds identified by HTS, and conduct comparative analysis to summarize their characteristics and application prospects.

##### E05657

Compound E05657 (2-fluorophenyl-N-6-methylpyridin-2-yl-5-trifluoromethyl-1H-pyrazole-4-carboxamide) is a compound capable of up-regulating the OPG/RANKL expression ratio, characterized by high activity and low effective concentration.

In June 2016, in a study by Gong et al. (2016), E05657 emerged as a small-molecule compound for treating osteoporosis from 8,160 compounds through HTS technology. In this study, Gong and colleagues established a two-stage cell model based on a dual reporter gene system. To identify potential upregulators of Osteoprotegerin (OPG) expression, a luciferase reporter-based UOP cell line was developed. This cell line served as a HTS platform, enabling the initial screening of 56 candidate compounds selected from a library of 8,160 small molecules. Subsequently, to validate the activity of the 56 positive compounds screened out in the first step, a UORP cell line was established for further characterization. Through this rigorous validation process, compound E05657 emerged as a promising candidate. Mechanistically, E05657 can be used to improve osteoporosis by up-regulating the OPG/RANKL ratio, inducing OPG secretion in osteoblasts, and inhibiting osteoclast differentiation (Gong et al., 2016). However, the in-depth mechanism of its regulation on OPG and RANKL expression and its *in vivo* therapeutic efficacy and safety still need to be further explored through *in vitro* and *in vivo* experiments, which is a key direction for its subsequent clinical transformation.

##### H5

H5 is a novel and effective Notum inhibitor with favorable drug-like properties. Previous research reports have indicated that Notum belongs to the α/β hydrolase superfamily and acts as a Wnt deacylase, inhibiting osteogenic differentiation by affecting the transduction of the downstream Wnt/β-catenin signaling pathway (Zhang et al., 2015; Yang et al., 2021).

In 2025, a research report by Liu et al. (2025a) proposed that this compound was screened out by HTS technology. The principle is based on the intramolecular charge transfer (ICT) mechanism between an electron donor (HTCF fluorophore) and an electron-withdrawing group (n-hexanoate) to develop a novel near-infrared (NIR) fluorescent probe (HX-HTCF). The probe was co-incubated with the target Notum enzyme, and the target compound was subsequently identified by detecting the degree of fluorescence signal attenuation after adding candidate compounds. Eventually, H5 was identified as a potent Notum inhibitor. In terms of mechanics, H5 activates the Wnt/β-catenin signaling pathway to significantly promote osteoblast differentiation and mineralization. Animal experiments have confirmed that H5 can effectively combat osteoporosis. Compared with traditional OP drugs, H5 has a clear target (Notum) and a novel mechanism of action, which avoids the safety risks of non-specific regulation of bone metabolism, showing good clinical transformation potential (Liu et al., 2025a). However, its long-term *in vivo* metabolic stability and potential off-target effects still need to be further evaluated.

##### Compound I and glabrone

Compound I and glabrone (GI, an isoflavone derivative) are two OP-related active compounds identified by HTS based on the same reporter gene assay system, which reflects the universality and flexibility of HTS in screening compounds with different structures but similar therapeutic directions.

Compound I, a 4-nitroimidazole derivative, is a small-molecule compound artificially synthesized by Dr. James Corte (Chen et al., 2006). In previous studies, Chen et al. (2006) constructed a cell-based reporter gene assay system. Combined with automated screening platform, this system uses the TRAP promoter to drive the luciferase reporter gene to systematically screen 100,000 small-molecule compounds. By monitoring the transcriptional and protein expression levels of the TRAP gene, the study evaluated the inhibitory effects of various compounds on the RANKL signaling pathway, and ultimately successfully identified Compound I with the strongest inhibitory activity. Mechanistically, it can selectively inhibit osteoclastogenesis by targeting the RANKL/RANK pathway, which can reduce excessive bone resorption in osteoporosis, and maintain bone mineral density. This suggests that Compound I may be a promising candidate for OP treatment, although its therapeutic efficacy and clinical significance *in vivo* still need to be further clarified (Chen et al., 2006). Based on the working principle of this system, Hojo et al. (2008) screened a new compound library and identified a novel osteogenic drug: the isoflavone derivative, GI. Unlike Compound I, which mainly inhibits osteoclast differentiation, glabrone focuses on promoting osteoblast activity, which reflects the diversity of HTS in screening compounds with different therapeutic targets. This also indicates that the integration of HTS with reporter gene systems can be flexibly adjusted according to the research focus, providing an efficient tool for multi-directional OP drug screening.

##### Baicalein

Baicalein, a flavonoid compound primarily extracted from the roots of *Scutellaria baicalensis* (Chinese skullcap), exerts multiple beneficial effects on human health (Moghaddam et al., 2014). It possesses potent anti-inflammatory, antioxidant, and antitumor activities (Li et al., 2022; Liu et al., 2021; Li et al., 2025b; Bie et al., 2017).

In June 2021, a study by Lee et al. (2021) further identified its bioactivity in bone repair. The researchers developed a drug screening model based on competitive binding between aptamers and antibodies to screen for sclerostin inhibitors. Ninety-six compounds were co-incubated with FAM-labeled aptamers, and six compounds were initially screened *via* fluorescence intensity detection. Subsequently, the screening results based on aptamers were compared with those of competitive immunoassays based on antibodies for further verification, and eventually baicalein, a potential inhibitor of sclerostin, was selected. Mechanistically, *in vitro* and *in vivo* experiments demonstrated that baicalein directly competes with sclerostin, activates the Wnt signaling pathway to promote osteoblastic differentiation, and thereby enhances bone formation (Lee et al., 2021). Compared with synthetic compounds (*e.g*., H5), baicalein, as a natural product, has higher safety and lower toxic and side effects, which is an advantage in clinical application. However, its low bioavailability and unstable *in vivo* metabolism are key issues that need to be solved in its subsequent research.

##### Daidzein

Daidzein, also known as daidzein, is a phytoestrogenic isoflavone primarily found in leguminous plants, which plays a significant role in the prevention and treatment of various diseases (Alshehri et al., 2021), including cancer, cardiovascular diseases, obesity, *etc*. (Mhone et al., 2022; Hong, Nam & Nam, 2023a; Crespillo et al., 2011).

In December 2009, using HTS technology, Li et al. (2009) conducted experiments and found that daidzein, as a small-molecule compound upregulating BMP2 expression, can be used for the treatment of OP. A BMP2 luciferase reporter system was constructed to monitor the transcriptional activity of the BMP2 gene in real time. Subsequently, luciferase luminescence intensity was employed to initially screen out five positive compounds from 734 natural compounds and 2,458 synthetic compounds. Three repeated experiments were performed on the initially screened positive compounds to confirm stable activity, and finally three positive compounds were identified: daidzein, genistein, and 2-acetyl-dibenzothiophene (2-ABT). Eventually, *in vitro* experiments have confirmed that daidzein showed the most advantages in promoting BMP2 expression (Li et al., 2009). The latest research has shown that daidzein also has a good anti-OP effect *in vivo* (Harahap et al., 2024). Similar to baicalein, daidzein is a natural product with high safety, but its weak activity and low bioavailability limit its clinical application, and structural modification or formulation optimization based on HTS technology is a feasible direction for its improvement.

##### ZINC000014951634 and ZINC000040976869

ZINC000014951634 (endorphin-2 analog) and ZINC000040976869 (tetrahydroxysqualene derivative) are two natural inhibitors targeting mitogen-activated protein kinase-activated protein kinase 2 (MAPKAPK2), which were identified through HTS combined with bioinformatics analysis and molecular simulation technology, reflecting the development trend of HTS towards intelligence and precision.

ZINC000014951634 and ZINC000040976869 are two natural inhibitors targeting mitogen-activated protein kinase-activated protein kinase 2 (MAPKAPK2), with their compound components being endorphin-2 analogs and tetrahydroxysqualene derivatives respectively. In addition, both of them have been predicted to have high safety and metabolic stability *in vivo*. In November 2022, Zhao et al. (2022) selected MAPKAPK2 as a key target for regulating osteoclast differentiation and bone resorption by combining bioinformatics analysis with previous research evidence that MAPKAPK2 maintains skeletal integrity in chemotherapy-treated mice by inhibiting the p38MAPK-MK2 axis (Yao et al., 2020). First, they obtained the crystal structure of MAPKAPK2 protein from the Protein Data Bank and prepared macromolecules, then constructed a ligand library with 18,225 compounds from the ZINC15 database and performed high-throughput virtual screening *via* the LibDock module in DS with LibDock scores as the detection method. 10,075 ligands docked with MAPKAPK2 were obtained. Next, pharmacological property prediction was performed on the top 30 candidate compounds to obtain five relatively safe compounds meeting the characteristics. Eventually, ZINC000014951634 and ZINC000040976869 were identified as ideal lead compounds targeting MAPKAPK2 through high-precision molecular docking and molecular dynamics simulation. Mechanistically, both can inhibit MAPKAPK2 kinase activity and synergistically regulate inflammatory and oxidative stress pathways to reverse the progression of OP (Zhao et al., 2022).

Compared with other compounds mentioned above, these two compounds are identified through virtual HTS combined with molecular simulation, which greatly reduces the screening cost and cycle, and their *in silico* predicted safety and metabolic stability provide a good foundation for subsequent *in vitro* and *in vivo* verification. However, their actual *in vivo* activity and toxic and side effects still need to be confirmed by experimental studies to promote their clinical transformation.

##### Comparative analysis of OP-related compounds screened by HTS

To further clarify the characteristics of OP-related compounds identified by HTS and provide guidance for subsequent research, this section conducts a critical comparative analysis of the above-mentioned compounds from the aspects of screening technology, target pathways, therapeutic direction, and advantages/disadvantages, focusing on the integration mode of HTS with high-end technologies and the differences in their mechanism of action.

In terms of screening technology integration, the compounds can be divided into three categories: (1) HTS combined with reporter gene systems (E05657, Compound I, glabrone, daidzein), which is the most traditional and widely used mode, with the advantages of high throughput and strong operability, and is suitable for large-scale initial screening of compounds; (2) HTS combined with fluorescent probe technology (H5), which improves the sensitivity and specificity of screening, especially suitable for the screening of enzyme inhibitors (*e.g*., Notum); (3) HTS combined with bioinformatics and molecular simulation (ZINC000014951634, ZINC000040976869), which realizes virtual screening, reduces the cost of experimental screening, and can predict the pharmacological properties of compounds in advance; (4) HTS combined with aptamer-antibody competitive binding (baicalein), which provides a new idea for the screening of target inhibitors with high specificity.

In terms of target signaling pathways, the compounds mainly act on four core pathways regulating bone metabolism: (1) OPG/RANKL pathway (E05657, Compound I), which mainly inhibits osteoclast differentiation and reduces bone resorption; (2) Wnt/β-catenin pathway (H5, baicalein), which mainly promotes osteoblast differentiation and enhances bone formation; (3) BMP2 pathway (daidzein), which focuses on regulating osteoblast activity to promote bone formation; (4) p38MAPK-MK2 pathway (ZINC000014951634, ZINC000040976869), which synergistically regulates inflammation and oxidative stress to improve OP. It is worth noting that the Wnt/β-catenin pathway is a common target pathway for H5 and baicalein, but the two compounds act on different targets (Notum *vs*. sclerostin) in the pathway, which reflects the diversity of HTS in screening compounds targeting different nodes of the same pathway, providing multiple options for OP therapy.

In terms of therapeutic direction, the compounds can be divided into two types: (1) Inhibiting bone resorption (E05657, Compound I, ZINC000014951634, ZINC000040976869), which is consistent with the mechanism of traditional bisphosphonates but has higher specificity and lower toxic and side effects; (2) Promoting bone formation (H5, baicalein, glabrone, daidzein), which makes up for the deficiency of traditional drugs that mainly inhibit bone resorption and cannot fundamentally improve bone mass. In addition, natural compounds (baicalein, daidzein) have the advantage of high safety, while synthetic compounds (E05657, H5, Compound I) have stronger activity and more controllable structure, and virtual screening compounds (ZINC000014951634, ZINC000040976869) have the advantage of low screening cost and high efficiency.

In summary, HTS technology, through integration with different high-end technologies, can screen OP-related compounds with diverse structures and mechanisms of action, which provides a rich basis for OP drug development. However, most of the current compounds are still in the preclinical research stage, and there are common problems such as unclear *in vivo* therapeutic efficacy, poor bioavailability, and potential off-target effects. Future research should focus on optimizing the integration mode of HTS with high-end technologies (*e.g*., combining single-cell sequencing with HTS to improve screening accuracy), conducting in-depth research on the *in vivo* mechanism of compounds, and optimizing their drug-like properties to accelerate their clinical transformation. At the same time, the comparative analysis of existing compounds can provide a reference for the design and screening of new OP drugs, avoiding redundant research and improving research efficiency.

#### Osteoarthritis (OA)

Osteoarthritis (OA) is a degenerative joint disease caused by degeneration and wear of articular cartilage, accompanied by synovial inflammation and osteophyte formation, which leads to joint pain and functional impairment (Tang et al., 2025) and affects the quality of life of patients. Current treatment methods have limitations. For example, oral non-steroidal anti-inflammatory drugs (NSAIDs) can relieve pain symptoms, however, long-term use may cause serious complications such as gastrointestinal bleeding (Tai & McAlindon, 2021). Joint replacement can restore joint function, but surgical trauma-induced tissue stress responses and limited service life due to prosthetic material wear often require secondary revision surgery (Sabah, Alvand & Price, 2021). New therapies such as stem cell therapy and gene editing also face challenges such as inconsistent efficacy and insufficient targeting (Kim, Choi & Kim, 2020). Against this background, the development of novel drugs and functional materials for OA relief and treatment is urgently needed. High-throughput screening (HTS) technology, by integrating with various advanced techniques (*e.g*., dual-reporter organoid systems, luminescence technology, fluorescence imaging), has become a powerful tool for efficiently discovering OA-related active compounds, which not only shortens the screening cycle but also improves the accuracy and targeting of candidate compound identification. The following sections detail the screening process, mechanism of action, and therapeutic potential of several OA-related active compounds identified by HTS-based integrated strategies, followed by a critical comprehensive analysis of their commonalities, differences in signaling pathways, and HTS methods.

##### Phentolamine

Phentolamine (PM) is a reversible, non-selective α-adrenergic antagonist. To date, it has been shown to have antiarrhythmic, antihypertensive, heart failure-improving, and erectile dysfunction-treating effects, and can be also used to enhance antibiotic activity (Degraff et al., 1976; Browne et al., 2016; Goldstein, 2000; Cui et al., 2023).

In 2024, Wei et al. (2024) established a human expanded pluripotent stem cell (hEPSC)-derived chondral organoid system carrying COL2A1-mCherry and COL10A1-eGFP dual reporters, enabling real-time dynamic monitoring of chondrogenic differentiation and hypertrophy. Using this model, they performed a two-step screening of 2,040 FDA-approved drugs. First, flow cytometry (FCM) was used to quantify the proportion of COL2A1+ cells, identifying eight chondrogenesis-promoting candidate compounds. Subsequently, fluorescence imaging and FCM were employed to evaluate the inhibitory effect on COL10A1+ cells, leading to the selection of phentolamine (PM), an α-adrenergic receptor (α-AR) antagonist, as the primary candidate. Mechanistically, PM can antagonize α2-adrenergic receptors (α2-ARs), block the cGMP/SLPI signaling axis to inhibit chondrocyte hypertrophy and degeneration, disrupt the HES1/RUNX2-mediated chondrocyte hypertrophy pathway, and upregulate SOX9 and ACAN to maintain chondrocyte homeostasis, thereby alleviating osteoarthritis. Further *in vivo* experiments confirmed the therapeutic potential of PM for osteoarthritis (Wei et al., 2024). It is worth emphasizing that one of the core advantages of this study lies in the in-depth integration and application of the 3D chondral organoid model and HTS technology: compared with 2D monolayer chondrocyte culture, the 3D organoid can better retain the phenotype of chondrocytes, accurately simulate the *in vivo* cartilage microenvironment, effectively avoid the defects of 2D culture, provide a more reliable *in vitro* model for HTS, and help efficiently screen out PM as a candidate drug; meanwhile, the combination of this 3D model and HTS technology not only successfully promoted the discovery of PM, but also provided a new paradigm for the HTS of OA therapeutic drugs, which can efficiently screen candidate drugs on the basis of simulating the pathological characteristics of OA, and accelerate the research and development process of OA therapeutic drugs.

##### Ethyl gallate

Ethyl gallate (EG) is a phenolic compound isolated from walnut kernels, Euphorbia plants, and Chinese gallnuts, functioning as a natural oxidative stress inhibitor. It exhibits various pharmacological activities, including anti-tumor, anti-HIV, anti-diabetic, and anti-obesity effects (Liu et al., 2019; Muddu Krishna et al., 2023; Ahn et al., 2022). Given that oxidative stress and ferroptosis are key pathological mechanisms of OA chondrocyte damage, EG was identified as a potential OA therapeutic compound through HTS targeting oxidative stress-induced chondrocyte injury, combined with a carrier delivery system to enhance its bioavailability.

In January 2025, Chen et al. (2025) employed a tert-butyl hydroperoxide (TBHP)-induced chondrocyte oxidative stress model to screen a library of over 600 natural compounds. Using the CCK-8 assay to assess cell viability, they identified EG as one of the top ten compounds capable of inhibiting oxidative stress-induced cell death. DCFH-DA fluorescence probe analysis confirmed EG’s superior reactive oxygen species (ROS) scavenging ability. Subsequent *in vitro* and *in vivo* experiments demonstrated that EG specifically inhibits ferroptosis. By encapsulating EG in a pH-responsive metal-organic framework (MOF) carrier (PZA), the researchers further enhanced its bioavailability and targeted therapeutic efficacy. This study not only identified EG as a promising OA candidate compound but also highlighted the value of HTS combined with nanocarrier technology in optimizing the therapeutic effect of active compounds. These findings highlight EG’s substantial potential for osteoarthritis (OA) management (Chen et al., 2025).

##### Panobinostat

Panobinostat is a first-line histone deacetylase inhibitor (HDACI) with potent anti-tumor activity. The European Commission has approved its combination with bortezomib and dexamethasone for treating multiple myeloma (Maouche et al., 2022; Van Veggel, Westerman & Hamberg, 2018). Additionally, it exhibits inhibitory effects against liver cancer, diffuse intrinsic pontine glioma, testicular tumors, and other malignancies (Monje et al., 2023; Lobo et al., 2020). Its potential in OA treatment was discovered through HTS targeting the FoxO1 transcription factor, which plays a key role in chondrocyte homeostasis and anti-inflammation.

In February 2023, Ohzono et al. (2023) uncovered its potential for osteoarthritis (OA) treatment. Using a luciferase reporter assay in human SW1353 chondrosarcoma cells harboring the FoxO1 promoter, they screened 11,948 clinically developed or preclinically validated compounds to identify small molecules activating the FoxO1 transcription factor. Initial screening yielded 56 hits, including 24 HDACIs. Dose-response analysis identified the four most potent HDACIs: Panobinostat, Vorinostat/SAHA, Givinostat/ITF2357, and Dacinostat/LAQ824. Subsequent *in vitro* and *in vivo* experiments confirmed that Panobinostat alleviates OA by activating the FoxO1/autophagy pathway and suppressing HDAC-mediated inflammatory responses (Ohzono et al., 2023). Both preclinical and clinical studies have demonstrated its efficacy in OA models, with low-dose regimens exhibiting favorable biosafety profiles. Future research should focus on validating these findings in spontaneous aging-related animal models to expedite clinical translation. This study demonstrates the advantage of HTS based on reporter gene assays in efficiently identifying target-specific small-molecule compounds.

##### Skatole (3-methylindole)

Skatole (3-methylindole) is a natural heterocyclic compound found in vertebrate feces, applied in the perfume industry and as an ice cream flavor additive, with its biological effects exhibiting duality (Zgarbová & Vrzal, 2023). Previous studies have shown that skatole induces death of intestinal epithelial cells (Kurata et al., 2019), while reports indicate that appropriate concentrations of skatole can protect hepatocytes (Hong et al., 2023b). Its OA-alleviating effect was identified through HTS targeting macrophage polarization, which is an important regulatory link in OA synovial inflammation.

Wang et al. (2025a) demonstrated that skatole alleviates OA by maintaining chondrocyte homeostasis. Focusing on the regulatory role of macrophage polarization in OA progression, using the RAW264.7 macrophage model and detecting the expression of the M2 macrophage marker Cd206 (Mrc1), they initially screened approximately 50 candidate compounds from the MCE (HY-L065) traditional Chinese medicine active compound library. The primary hits were validated and optimized *via* RT-qPCR and Western blot, with skatole identified as the most effective modulator. Mechanistically, skatole regulates macrophage polarization and metabolism through multiple pathways to alleviate osteoarthritis (Wang et al., 2025a). Its function has been validated in an *in vivo* OA model.

##### Vitamin K

Vitamin K (VK), a fat-soluble vitamin, is approved for preventing neonatal hemorrhagic diseases. It includes natural plant- and animal-derived forms (K1 and K2) and synthetic homologs (K3 and K4), with different forms potentially exhibiting distinct biological functions (Mladěnka et al., 2022). Previous reports have shown that VK can improve osteoporosis by regulating osteoblastogenesis and osteoclastogenesis *via* the nuclear factor κB (NF-κB) signaling pathway (Fusaro et al., 2020). In recent years, its potential to ameliorate osteoarthritis (OA) has also been increasingly recognized.

In August 2024, Shi et al. (2024) established a stable and reliable ferroptosis induction model by treating chondrocytes with different concentrations of GPX4 inhibitors or system Xc^−^ inhibitors to determine optimal conditions for inducing chondrocyte ferroptosis. Using this model, they co-treated chondrocytes with 1,527 FDA-approved candidate drugs capable of inhibiting ferroptosis and GPX4/system Xc^−^ inhibitors, quantifying the effect of each drug on ferroptosis *via* the modulation effect (Me) index. Initial screening identified 11 drugs that effectively resisted ferroptosis in RSL3- or erastin-induced chondrocytes. Among these, VK1 demonstrated the most potent efficacy in both RSL3 and erastin groups. Mechanistic studies confirmed through *in vitro* and *in vivo* experiments that VK enhances Gas6 expression, further promoting Gas6/AXL signaling and the downstream PIK3/AKT pathway to inhibit RSL3-induced ferroptosis in chondrocytes (Shi et al., 2024). This study not only identified VK as a promising OA candidate compound but also established a standardized HTS method for screening anti-ferroptosis compounds in OA.

##### Mocetinostat

Mocetinostat, a novel isotype-selective HDAC enzyme also known by its chemical name MGCD0103, was approved for phase II trials in Hodgkin lymphoma in 2010 (Khathayer & Mikael, 2024). Recent studies have also revealed its potential for treating osteoarthritis (OA). Similar to Panobinostat, it belongs to HDACIs, but its potential in OA treatment was discovered through HTS targeting the KLF4 transcription factor, which is closely related to chondrocyte differentiation and cartilage homeostasis.

In April 2023, Kawata et al. (2023) established a KLF4 reporter cell line in SW1353 human chondrosarcoma cells using HiBiT luminescence technology. They screened 11,948 compounds from the ReFRAME library to identify small molecules capable of activating KLF4, initially identifying 51 hits. After repeated screening to exclude false positives, 18 compounds with stable KLF4 activation ability were identified. Functional validation of these 18 compounds on SW1353 cells revealed that three class I HDAC inhibitors significantly upregulated chondrogenesis-related gene expression, with Mocetinostat exhibiting the most prominent biological activity. Mechanistic studies confirmed through *in vitro* and *in vivo* experiments that Mocetinostat maintains cartilage homeostasis and treats OA by activating the KLF4/KLF2-FOXO1 axis (Kawata et al., 2023). This study also demonstrates the advantage of HiBiT luminescence-based HTS in improving the efficiency and accuracy of candidate compound screening.

##### Comparative analysis of OA-related compounds screened by HTS

To further clarify the characteristics of OA-related compounds identified by HTS and guide subsequent research, this section provides a critical comparative analysis focusing on HTS integration modes and mechanisms of action.

In terms of screening technology integration, the compounds fall into four categories: (1) HTS combined with dual-reporter organoid system (Phentolamine): integrates 3D organoid modeling and real-time dual-reporter monitoring, overcoming 2D culture limitations with high reliability and clinical relevance; (2) HTS combined with oxidative stress/ferroptosis model and fluorescence detection (Ethyl Gallate): targets chondrocyte oxidative damage, simple and specific, and combined with nanocarriers enhances therapeutic efficacy; (3) HTS combined with luminescence reporter systems (Panobinostat, Mocetinostat): both use highly sensitive reporter assays to screen transcription factor modulators, demonstrating the advantage of reporter-based HTS; (4) HTS combined with macrophage model and molecular validation (Skatole): focuses on synovial inflammation and macrophage polarization, with RT-qPCR/Western blot validation ensuring reliability.

In terms of target signaling pathways, the compounds mainly act on four core pathways: (1) Chondrocyte hypertrophy-related pathways (Phentolamine targets α2-AR/cGMP/SLPI and HES1/RUNX2); (2) Ferroptosis-related pathways (Ethyl Gallate *via* ROS scavenging, Vitamin K *via* Gas6/AXL/PIK3/AKT); (3) HDAC-related pathways (Panobinostat activates FoxO1/autophagy, Mocetinostat regulates KLF4/KLF2-FOXO1 axis); (4) Macrophage polarization-related pathways (Skatole promotes M1-to-M2 switching). Notably, HDAC is shared by Panobinostat and Mocetinostat, while ferroptosis is targeted by both Ethyl Gallate and Vitamin K, reflecting HTS versatility in screening compounds targeting different nodes of the same pathway or pathological process.

In terms of therapeutic direction, the compounds can be divided into two types: (1) Protecting chondrocytes (Phentolamine inhibits hypertrophy, Ethyl Gallate and Vitamin K inhibit ferroptosis, Panobinostat and Mocetinostat inhibit apoptosis); (2) Inhibiting synovial inflammation (Skatole regulates macrophage polarization, Panobinostat and Mocetinostat suppress HDAC-mediated inflammation). Natural compounds (Ethyl Gallate, Skatole, Vitamin K) offer high safety but Ethyl Gallate has poor bioavailability; repurposed drugs (Phentolamine, Panobinostat, Mocetinostat) have known safety profiles and can shorten clinical translation, though long-term OA safety needs confirmation; HDAC inhibitors have novel mechanisms, with Mocetinostat being isotype-selective and potentially offering higher specificity.

In summary, HTS integrated with diverse high-end technologies (organoid systems, reporter assays, fluorescence detection, macrophage models) enables discovery of structurally and mechanistically diverse OA compounds, enriching drug development. However, most candidates remain preclinical, facing challenges such as poor bioavailability of natural compounds (Ethyl Gallate), unclear optimal dosing and long-term safety (Skatole), need for aging-model validation (Panobinostat), and off-target risks (Mocetinostat, Phentolamine). Future research should optimize HTS integration modes (*e.g*., combining 3D organoid models with single-cell sequencing), improve natural compound bioavailability *via* delivery system modification, and deepen *in vivo* mechanistic studies to accelerate clinical translation. Meanwhile, comparative analysis of existing compounds can inform new OA drug design, avoiding redundancy and improving efficiency.

#### Bone defect and bone tumor

Bone defects and bone tumors are two major types of bone diseases that seriously threaten human skeletal health. They have distinct pathological characteristics but both have an urgent demand for the efficient discovery of therapeutic materials and drugs. The core focus of this study is to elaborate on how HTS technology can more effectively discover disease targets and screen active compounds/materials through its combination with a series of advanced technologies. Bone defect refers to partial or complete loss of bone tissue caused by trauma, infection, tumor resection, or congenital diseases, characterized by disruption of bone structural continuity and mechanical integrity (Wu et al., 2025; Shen et al., 2024; Wang et al., 2025b). Current treatments mainly include surgical and non-surgical approaches, with surgical interventions—such as bone grafting and implantation of artificial bone substitutes—being the most common methods for bone defect repair (Xue et al., 2022). Although bone grafting is widely used in clinical practice, it has limitations: large-scale bone defects exhibit low regeneration efficiency and high necrosis rates with traditional grafting, and existing implant materials often fail to simultaneously meet requirements for mechanical support, biological activity, and controllable degradation (Schemitsch, 2017; Zhang et al., 2025). Bone tumors are abnormal proliferative lesions occurring in bones or accessory bone tissues such as cartilage, bone marrow, and periosteum. The most common primary malignant bone tumors include osteosarcoma and Ewing sarcoma (Simpson & Brown, 2018). Both osteosarcoma and Ewing sarcoma predominantly affect adolescents and children and are highly aggressive. Due to the current lack of targeted therapeutic drugs, most patients undergoing conventional treatments require radiotherapy, chemotherapy, or limb amputation (Liu et al., 2025b). Amputation not only leads to disability but also causes depression and anxiety in patients. Therefore, the development of materials and drugs for bone defect treatment, as well as targeted agents for bone tumors, holds immense significance. HTS technology, when combined with advanced techniques, can expedite this process by circumventing the constraints of conventional low-throughput screening.

##### 1-S2-FT01

1-S2-FT01 is a copolymer composed of the monomer 1, 3, 5-triacyl-1, 3, 5-triazine-2, 4, 6 (1H, 3H, 5H)-trione (TATATO) and pentaerythritol tetrakis (3-mercaptopropionate) (PETMP), exhibiting excellent compressive and tensile properties.

Kavya Ganabady and her team successfully synthesized 90 polymers by leveraging thiol-ene click chemistry technology and employing ultraviolet (UV) rapid curing processes. Subsequently, they established a HTS platform by integrating polymer microarray technology with fluorescence microscopy and high-sensitivity detection methods (*e.g*., ImageJ software), introducing the 90 polymers into microarray plates for high-throughput preliminary screening. The researchers captured images using fluorescence microscopy and accurately measured the cell adhesion rate with the aid of high-sensitivity detection methods, such as ImageJ software. After rigorous screening, only approximately 18 polymers with outstanding cell adhesion rates advanced to the re-screening stage.

During the re-screening phase, the team systematically evaluated the material properties through mechanical property tests and cytotoxicity assays, eliminating materials with subpar performance or potential toxicity risks one by one. Eventually, three candidate materials emerged and entered the functional verification stage. The researchers focused on identifying their osteogenic differentiation ability and 3D scaffold adhesion. The results showed that the copolymer 1-S2-FT01, composed of TATATO and PETMP, was determined as the optimal candidate material due to its high bonding strength and excellent biocompatibility.

This study indicates that, in *in vitro* cell line experiments, 1-S2-FT01 exhibits potential value in promoting bone repair. However, its actual efficacy *in vivo* still requires further verification to accelerate the clinical translation of this material. Notably, this research establishes a typical HTS-based material screening paradigm: integrating material synthesis technology, microarray-based high-throughput screening, and high-sensitivity quantitative detection, which not only improves the efficiency of bone repair material screening but also ensures the reliability of screening results—providing a reference for subsequent HTS applications in bone tissue engineering materials.

##### Leflunomide (Lef)

Leflunomide (Lef), an isoxazole derivative, serves as a novel immunomodulator with potent anti-inflammatory effects and has been approved for the treatment of rheumatoid arthritis (Silverman et al., 2005). Beyond this, Lef exhibits numerous other biological actions, such as treating atherosclerosis and alleviating obesity (Jiang et al., 2024; Ji et al., 2023).

In a study by Fukuyasu et al. (2022), it was discovered that Lef also possesses the ability to promote new bone formation *in vivo*. Using a dual screening system, the researchers first screened the Lopack1280 compound library by detecting changes in GFP fluorescence intensity, identifying 1,030 potential candidate compounds. In the secondary screening, 11 compounds with significantly activated alkaline phosphatase (ALP) activity were selected through ALP activity assays. Cross-validation ultimately identified leflunomide (Lef), LFM-A13 (LFM), and 1-(5-isoquinolinesulfonyl)-3-methylpiperazine dihydrochloride (1-5) as potent candidate drugs. Through highly sensitive detection techniques, including quantitative PCR, WST-1 and CytoTox-Glo assays, and ALP/von Kossa staining, Lef was confirmed as the drug with the strongest osteogenic activity. Mechanistically, Lef promotes bone regeneration through multiple pathways, including activating the JAK/STAT signaling pathway, inhibiting proteasome activity, regulating immune cell functions, and influencing cell proliferation and differentiation (Fukuyasu et al., 2022). Fukuyasu et al. (2022) have validated Lef’s ability to promote cranial defect repair in rat models. Future research may focus on primate model experiments and human trials using high-throughput screening (HTS) technology to accelerate clinical translation.

##### 6,8-Dimethyl-3-(4-phenyl-1-imidazol-5-yl)quinolin-2(1H)-one

6,8-Dimethyl-3-(4-phenyl-1-imidazol-5-yl)quinolin-2(1H)-one (DIPQUO) is a small-molecule compound identified through high-throughput screening (HTS) integrated with a multi-technology detection platform, which ensures the accuracy and efficiency of candidate compound screening. In 2019, based on C2C12 mouse myoblasts, Cook et al. (2019) established a screening platform, which is integrated with highly sensitive detection techniques such as fluorescence emission assays, ALP staining assays, flow cytometry, high-pressure liquid chromatography and mass spectrometry for purity determination, and carbon and proton nuclear magnetic resonance (NMR) spectroscopy. This multi-technology integration addresses the limitations of single detection methods, enabling comprehensive evaluation of compound activity, purity, and structural characteristics in a high-throughput manner. After screening over 47,000 small-molecule compounds, DIPQUO was identified as a molecule that promotes ALP activation. Mechanistically, DIPQUO functions by activating the p38 MAPK signaling pathway to promote osteogenic differentiation, although its direct target remains unclear. Therefore, DIPQUO shows potential in bone repair (Cook et al., 2019).

Compared with the HTS strategies used for 1-S2-FT01 and Lef, this study highlights the advantage of integrating HTS with structural characterization technologies (HPLC, MS, NMR), which not only screens for active compounds but also clarifies their structural properties—laying a foundation for subsequent mechanism research and structural modification. In the future, combining gene editing technologies (*e.g*., CRISPR) to target and regulate key repair genes could further clarify its mechanism of action, thereby enabling more effective development of drugs for improving bone defects.

##### Mithramycin A (MTA)

Mithramycin A (MTA), also known asplicamycin, is a golden yellow acidic polyketide antibiotic produced by various Streptomyces species (Portugal, 2024). Widely used as an antitumor antibiotic, it has been applied in the treatment of breast cancer, hematological tumors, and other malignancies (Portugal, 2024). Additionally, it exhibits functions such as cardioprotection, neuroprotection, and maintenance of cartilage homeostasis (Geng et al., 2021; Osada et al., 2013; Choi & Choi, 2018). Previous studies have also identified MTA’s biological function in combating bone tumors, and its targeted anti-Ewing sarcoma activity—particularly suitable for early-stage bone tumors—has been further validated through HTS integrated with advanced reporter gene technology, which aligns with the therapeutic demand for precise targeting in early bone tumor treatment.

In June 2011, Grohar et al. (2011) constructed a reporter system by inserting the promoter of the EWS-FLI1 downstream target gene NR0B1 into a luciferase gene-containing vector and transfecting it into the Ewing sarcoma TC32 cell line. This reporter gene-based HTS platform is specifically designed for early-stage bone tumors, as it can specifically monitor the activity of the EWS-FLI1 oncogenic pathway (the key driver of Ewing sarcoma), enabling high-throughput screening of pathway-specific targeted compounds and avoiding non-specific cytotoxicity that may damage normal tissues. Using NR0B1-Luc luciferase expression and cell viability as detection criteria, they initially screened over 6,500 compounds isolated from natural product extracts, identifying 150 candidate compounds. To further improve screening specificity—critical for early-stage bone tumor therapy to reduce off-target effects—they optimized the HTS workflow through concentration gradient validation and log_2_ differential sum scoring, prioritizing compounds that specifically inhibit NR0B1 expression rather than simply inducing tumor cell death. Subsequently, multiplex PCR detection was used to verify the inhibitory effects of candidate compounds on multiple EWS-FLI1 downstream target genes, ultimately selecting mithramycin A as the lead compound.

Mechanistically, both *in vitro* and *in vivo* experiments have demonstrated that MTA can inhibit the expression of EWS-FLI1 downstream targets and induce DNA damage, thereby suppressing the growth of Ewing sarcoma (Grohar et al., 2011). Notably, MTA exerts its anti-tumor effect by targeting the EWS-FLI1 signaling pathway, which is a typical pathway-specific regulatory mode—consistent with the HTS screening strategy based on the reporter gene system. The currently screened MTA has entered the clinical trial phase, and its historical clinical application basis (in other malignancies) further supports its rapid clinical translation for early-stage bone tumors, fully demonstrating the advantage of HTS integrated with reporter gene technology in discovering targeted anti-tumor compounds for early-stage bone tumors.

##### Doxorubicin (DOX)

Doxorubicin (DOX), a potent topoisomerase II inhibitor, prevents DNA replication in cancer cells and was the first clinically approved chemotherapeutic drug for treating malignant tumors such as osteosarcoma, transplantable leukemia, and lymphoma (Almajidi et al., 2023). As a classic chemotherapeutic agent, DOX is mainly suitable for middle and advanced bone tumors (characterized by local invasion, distant metastasis, or unresectable tumors), where the priority is to inhibit tumor progression and control disease spread. Despite significant advancements in cancer treatment with DOX, its narrow therapeutic window poses challenges: suboptimal concentrations reduce efficacy, while excessive concentrations exacerbate side effects (*e.g*., cardiotoxicity, myelosuppression) (Fang et al., 2019; Amalina et al., 2023). Therefore, selecting appropriate drug concentrations is critical to balancing therapeutic efficacy and safety.

In August 2021, Tang et al. (2021) used a Christmas tree-structured HA-PDMS microfluidic chip (an advanced high-throughput platform with the advantages of low sample consumption, precise concentration gradient control, and high throughput) to screen DOX and determine its half-maximal inhibitory concentration (IC_50_) against osteosarcoma cells. This microfluidic-based HTS platform is particularly suitable for middle and advanced bone tumors, as it can efficiently optimize the therapeutic concentration of chemotherapeutic drugs, solving the clinical dilemma of narrow therapeutic window of DOX and providing a scientific basis for clinical dosage adjustment. First, UV spectrophotometry was used to scan DOX solutions and detect absorbance in different cell culture chambers, determining the concentration distribution of DOX within the chip. Second, ImageJ software was employed to count cells in various chambers, and changes in cell numbers were measured to assess DOX’s inhibitory effect on cell growth. This analysis inferred that the IC_50_ of DOX for UMR-106 cells was approximately 0.2 μg/mL (Tang et al., 2021).

Notably, the HTS strategy for DOX focuses on concentration optimization rather than new compound discovery, which is fundamentally different from the HTS paradigm for MTA. This difference is closely related to the pathological characteristics of different bone tumor stages: early-stage bone tumors require targeted compound discovery to achieve precise treatment, while middle and advanced bone tumors need to optimize the dosage of existing chemotherapeutic drugs to improve safety and efficacy—reflecting the flexibility of HTS technology in adapting to different clinical needs of bone tumor staging.

##### Comparative analysis of bone defect and bone tumor-related candidates identified by HTS

To further clarify the characteristics of bone defect and bone tumor-related candidates identified by HTS and provide guidance for subsequent research, this section conducts a critical comparative analysis of the above-mentioned materials and compounds from the aspects of screening technology, target pathways, therapeutic direction, and advantages/disadvantages, focusing on the integration mode of HTS with high-end technologies and the differences in their mechanism of action.

In terms of screening technology integration, the candidates can be divided into five categories: HTS combined with polymer microarray and high-sensitivity imaging detection (1-S2-FT01), which integrates thiol-ene click chemistry, UV rapid curing, fluorescence microscopy and ImageJ quantitative analysis. This mode establishes a standardized material screening paradigm with high throughput and good operability, and is suitable for high-throughput screening and performance evaluation of bone tissue engineering scaffold materials; HTS combined with dual fluorescence screening and multi-index functional verification (Leflunomide), relying on GFP fluorescence intensity primary screening and ALP activity secondary screening, combined with qPCR, WST-1, CytoTox-Glo assay and histological staining for cross validation. It can effectively eliminate false-positive compounds and is applicable to large-scale osteogenic compound library screening; HTS combined with multi-technology structural characterization platform (DIPQUO), integrated with fluorescence emission assay, ALP staining, flow cytometry, HPLC-MS and NMR spectroscopy. This method achieves simultaneous evaluation of compound activity, purity and structural characteristics, laying a foundation for subsequent mechanism research and structural modification; HTS combined with reporter gene system (Mithramycin A), constructed EWS-FLI1 pathway-specific luciferase reporter platform, combined with concentration gradient validation and multiplex PCR optimization. It features high screening specificity, avoids non-specific cytotoxicity, and is ideal for targeted compound screening of early bone tumors; HTS combined with microfluidic chip platform (Doxorubicin), adopts HA-PDMS microfluidic chip to build high-throughput concentration gradient system, combined with UV spectrophotometry and cell counting for IC50 determination. It is mainly used for dosage optimization of existing chemotherapeutic drugs rather than new compound discovery.

In terms of target signaling pathways, these candidates mainly act on core pathways related to osteogenic differentiation and bone tumor progression: Osteogenic metabolism-related pathways: Leflunomide mainly activates the JAK/STAT pathway, inhibits proteasome activity and regulates immune cell function to promote bone regeneration; DIPQUO functions by activating the p38 MAPK pathway to enhance osteogenic differentiation; 1-S2-FT01 improves cell adhesion and osteogenic capacity by optimizing material mechanical properties and biocompatibility without targeting classic signaling pathways; Bone tumor-related pathways: Mithramycin A specifically targets the EWS-FLI1 oncogenic pathway, inhibits downstream gene expression and induces DNA damage to suppress Ewing sarcoma growth; Doxorubicin inhibits topoisomerase II activity and blocks tumor cell DNA replication to restrain osteosarcoma proliferation. It is worth noting that HTS can screen materials or compounds targeting different pathological links of bone defects and bone tumors, reflecting the flexibility of HTS in adapting to different disease mechanisms.

In terms of therapeutic direction, the candidates can be divided into two types:

Promoting bone defect repair (1-S2-FT01, Leflunomide, DIPQUO). Among them, 1-S2-FT01 serves as artificial bone scaffold material for mechanical support and bone regeneration; Leflunomide and DIPQUO are small-molecule compounds that promote osteoblast differentiation, making up for the deficiency of traditional bone grafts with low regeneration efficiency; Intervening bone tumor progression (Mithramycin A, Doxorubicin). Mithramycin A is applied to targeted therapy of early Ewing sarcoma to achieve precise treatment and reduce amputation rate; Doxorubicin is used for chemotherapy of middle and advanced bone tumors to inhibit tumor invasion and metastasis. In addition, synthetic polymer material (1-S2-FT01) has controllable structure and excellent mechanical properties; repurposed clinical drugs (Leflunomide, Mithramycin A) have clear safety background and short clinical transformation cycle; newly screened small-molecule compounds (DIPQUO) have novel action mechanisms; microfluidic HTS optimized chemotherapeutic drugs (Doxorubicin) can balance efficacy and toxic side effects.

In summary, HTS technology, through integration with different high-end technologies, can screen diverse biomaterials and small-molecule compounds for bone defect and bone tumor treatment, which provides a rich basis for the development of orthopedic therapeutic agents. However, most of the current candidates are still in the preclinical research stage, and there are common problems such as insufficient *in vivo* efficacy verification, unclear direct target of active compounds, narrow tumor adaptation range, and unavoidable inherent side effects of chemotherapeutic drugs. Future research should focus on optimizing the integration mode of HTS with high-end technologies (*e.g*., combining 3D organoid model with HTS to improve screening simulation), conducting in-depth research on *in vivo* mechanism of candidates, and optimizing material properties and drug-like characteristics to accelerate their clinical transformation. At the same time, the comparative analysis of existing candidates can provide a reference for the design and screening of new bone repair materials and anti-bone tumor drugs, avoiding redundant research and improving research efficiency.

#### Fibrodysplasia ossificans progressiva

Fibrodysplasia ossificans progressiva (FOP) is a rare but destructive autosomal dominant genetic disorder characterized by extra-skeletal bone formation. Over 95% of FOP cases are caused by the R206H mutation in the ACVR1 gene (Activin A Receptor Type I), which encodes a key receptor in the bone morphogenetic protein (BMP) signaling pathway. This mutation leads to abnormal activation of the ACVR1 receptor, ultimately triggering heterotopic ossification (HO) in the absence of BMP ligands (Chaikuad et al., 2012; Convente et al., 2018). Typical features include great toe malformation and progressive HO in specific spatial patterns (Kaplan et al., 2008). Current available interventions mainly rely on hormone therapy and symptomatic treatment, and no curative strategy that can fundamentally block the pathological progression of FOP has been established yet. Against this background, the integration of HTS with reporter gene assay and cellular phenotypic evaluation is expected to serve as a promising technical route to accelerate the discovery of candidate small molecules and therapeutic targets for FOP intervention.

##### Dipyridamole (dipyridamole, Dipy)

Initially used for treating angina, Dipy—a FDA-approved drug—has demonstrated multiple therapeutic functions, including antithrombotic, anti-inflammatory, vasodilatory effects, enhancing cytotoxicity of anticancer drugs, and regulating myogenesis (Rogosnitzky et al., 2016; Eisert, 2012; Tan, Heng & Mak, 2019; Xue, Ma & Liu, 2019; Marco-Bonilla et al., 2023; Abdelghany et al., 2021).

In 2016, Cappato et al. (2016) developed a high-throughput screening system using an ACVR1 promoter-luciferase reporter and identified that Dipy significantly reduces heterotopic ossification by inhibiting ACVR1 gene expression and BMP signaling. First, they established a stable ATDC5 cell line (mouse chondrogenic cells) expressing a luciferase reporter driven by the 2.9 kb ACVR1 promoter region to quantify ACVR1 transcriptional activity. Using this cell line, they screened 1,280 FDA-approved compounds from the Prestwick Chemical Library and identified 18 ACVR1 promoter inhibitors. Among them, Dipy treatment reduced luciferase activity by over 0.4-fold (*via* ONE-Glo™ reagent assay), indicating the most significant inhibition. *In vitro* and *in vivo* experiments further validated Dipy’s effect in reducing heterotopic ossification (Cappato et al., 2016). Additionally, CellTiter-Fluor staining showed low toxicity of Dipy, suggesting that future research can focus on animal model studies and human trials to accelerate clinical translation.

#### Rheumatoid arthritis (RA)

Rheumatoid arthritis (RA) is a chronic autoimmune disease affecting multiple joints, characterized by clinical manifestations such as joint pain, swelling, stiffness, and severe cases leading to joint deformity and functional impairment (Ngian, 2010). Its pathogenesis is complex, involving genetic, environmental, and immune factors. Traditional therapeutic drugs include non-steroidal anti-inflammatory drugs and hormones. In recent years, disease-modifying antirheumatic drugs (DMARDs) such as methotrexate, sulfasalazine, infliximab, etanercept, abatacept, and rituximab have been increasingly used for treatment and prevention of RA (Lin, Anzaghe & Schülke, 2020). Despite significant advancements in RA treatment, current approaches still have limitations. For example, biologic and targeted synthetic DMARDs are costly and may cause severe side effects such as infections, liver damage, and cytopenia (Aletaha & Smolen, 2018; Brown, Pratt & Hyrich, 2024). Against the backdrop of unmet clinical needs, the combined application of high-throughput phenotypic screening, virtual screening and computational simulation technologies serves as a promising research strategy to explore novel lead compounds and potential therapeutic targets for RA.

##### Berberine

Berberine (BBR) is an isoquinoline alkaloid isolated from the rhizomes of *Coptis chinensis*, renowned for its significant health benefits (Feng et al., 2019). It exhibits activities such as lipid-lowering, hypoglycemic, anti-inflammatory, and antitumor effects (Chen et al., 2022; Araj-Khodaei et al., 2024; Lu, Fu & Li, 2022; Feng et al., 2024). Additionally, previous studies have indicated BBR’s potential in treating osteoporosis and osteoarthritis (Zhang, Ma & Zhang, 2021). In recent years, its therapeutic potential for RA has also been gradually uncovered.

In 2024, Zhang et al. (2024) constructed a TNF-α-induced inflammatory RA cell pathological model based on human fibroblast-like synoviocyte MH7A, and further established a cell phenotype-based high-throughput screening workflow. Subsequently, they screened 2,661 natural product compounds in this model using the Cell Counting Kit-8 (CCK-8) to detect cell viability, aiming to identify compounds affecting the viability of RA-FLS cells. BBR was ultimately selected as the most promising compound. Mechanistically, *in vitro* experiments have demonstrated that BBR activates the Hippo signaling pathway to inhibit the pro-inflammatory and migratory capabilities of RA-FLS cells, thereby regulating pyroptosis and inflammation. *In vivo* experiments verified that BBR downregulates the expression of the NLRP3 inflammasome in synovial tissues while activating the Hippo pathway to alleviate joint symptoms (Zhang et al., 2024). Currently, BBR has been confirmed as an ideal compound with therapeutic potential for RA.

##### F3139-1037

F3139-1037 (C_25_H_19_ClO_6_) is a natural compound that co-targets COX-2 and JAK1, featuring excellent *in vivo* transport capacity, pharmacokinetic adaptability, and drug development potential.

In March 2025, Different from the cell phenotypic HTS strategy of berberine, Maji (2025) adopted a computer-aided virtual high-throughput screening strategy integrated with multiple computational high-end technologies to mine potential anti-RA candidates from a natural compound library containing 3,666 components. First, molecular docking software (AutoDock Vina) and docking energy scores were used to evaluate the binding affinity of each compound to COX-2 and JAK1, preliminarily screening out 10 candidate compounds. Second, the SwissADME tool was employed to analyze the drug-likeness of the candidates, and DataWarrior was used to predict toxicity, eliminating compounds with strong side effects. Finally, molecular dynamics simulations were performed to validate their stability (Maji, 2025). In summary, F3139-1037 stood out due to its high affinity for dual targets, superior pharmacokinetic properties, and low toxicity, emerging as a potential candidate for treating RA.

##### Comparative analysis of RA-related compounds identified by HTS

To further clarify the characteristics of RA-related compounds identified by HTS and provide guidance for subsequent research, this section conducts a critical comparative analysis of the above-mentioned compounds from the aspects of screening technology, target pathways, therapeutic direction, and advantages/disadvantages, focusing on the integration mode of HTS with high-end technologies and the differences in their mechanism of action.

In terms of screening technology integration, the compounds can be divided into two categories: (1) HTS combined with cell phenotypic pathological model (Berberine, BBR), which is a conventional phenotypic screening mode with the advantages of high throughput and strong operability, and is suitable for large-scale initial screening of natural product compound libraries; (2) HTS combined with bioinformatics and molecular simulation (F3139-1037), which realizes computer-aided virtual screening, reduces the cost of experimental screening, and can predict drug-likeness, toxicity and molecular binding stability of compounds in advance. In terms of target signaling pathways, the compounds mainly act on core pathways regulating RA inflammatory response: (1) Hippo/NLRP3 inflammasome pathway (BBR), which inhibits the inflammatory activation and migration of RA-FLS cells, regulates cell pyroptosis and alleviates synovial inflammation; (2) Dual COX-2/JAK1 inflammatory pathway (F3139-1037), which simultaneously targets two key inflammatory nodes to exert synergistic anti-RA efficacy. It is worth noting that both BBR and F3139-1037 are used for RA intervention, but the two compounds act on different inflammatory regulatory pathways, which reflects the diversity of HTS in screening compounds targeting different pathological nodes of RA, providing multiple options for RA therapy.

In terms of therapeutic direction, the compounds can be divided into one unified type: ameliorating RA inflammatory injury and joint pathological changes, which makes up for the deficiency of traditional DMARDs such as high price and obvious adverse reactions. In addition, natural monomer compounds (BBR) have the advantage of high safety and abundant natural sources, while virtual screened natural compounds (F3139-1037) have the advantages of dual-target binding activity, favorable pharmacokinetic performance and low screening cost and high efficiency.

In summary, HTS technology, through integration with different high-end technologies, can screen RA-related compounds with diverse structures and mechanisms of action, which provides a rich basis for RA drug development. However, most of the current compounds are still in the preclinical research stage, and there are common problems such as insufficient *in vivo* mechanism verification, unclear long-term biosafety, and lack of systematic pharmacological verification. Future research should focus on optimizing the integration mode of HTS with high-end technologies (*e.g*., combining 3D organoid model with HTS to improve screening accuracy), conducting in-depth research on the *in vivo* mechanism of compounds, and optimizing their drug-like properties to accelerate their clinical transformation. At the same time, the comparative analysis of existing compounds can provide a reference for the design and screening of new RA drugs, avoiding redundant research and improving research efficiency.

## Discussion

Currently, HTS has been widely integrated with microfluidics, 3D bioprinting, organoid models, artificial intelligence and multi-omics technologies. It has evolved into a core approach for exploring disease mechanisms, identifying therapeutic targets, and discovering small-molecule drugs and biomaterials in orthopedic research. This review systematically summarizes the applications of HTS across osteoporosis, osteoarthritis, bone defects, bone tumors, fibrodysplasia ossificans progressiva, and rheumatoid arthritis, and consolidates a broad range of candidate compounds, natural products, repurposed clinical drugs, and tissue-engineered biomaterials. Existing evidence demonstrates that HTS enables effective screening of molecules governing pivotal signaling pathways, including Wnt/β-catenin, BMP2, OPG/RANKL and p38MAPK. It also targets key pathological processes such as ferroptosis and inflammation, forming a relatively complete technical workflow from library construction to functional validation. Even so, most published studies remain predominantly descriptive. Many merely report screening outcomes without in-depth mechanistic interpretation, horizontal comparison, or systematic generalization. Moreover, the inherent technical limitations and translational bottlenecks of HTS in orthopedic research have not been sufficiently elaborated in previous reviews.

In terms of simulation authenticity, most current HTS studies rely on conventional two-dimensional cell cultures or simplified 3D models. These systems cannot fully recapitulate the native bone microenvironment, which comprises multiple cell populations, vascular networks, nerve innervation, and dynamic mechanical cues. Although 3D organoid and bioprinting technologies have advanced considerably, most constructs remain tissue-specific and lack vascularization and immune integration. Such oversimplification inevitably elevates false-positive and false-negative rates. Many compounds exhibit promising activity in simple *in vitro* models but fail to maintain stable efficacy in animal studies and preclinical settings. Additionally, most screening assessments depend on single phenotypic readouts, ignoring crosstalk between signaling pathways and dynamic cellular interactions, making it difficult to accurately evaluate long-term safety and comprehensive pharmacological profiles of candidate agents.

In terms of screening strategies and library resources, HTS still faces notable bottlenecks in orthopedic research. Existing libraries mainly consist of approved pharmaceuticals, synthetic compounds and limited natural products. Few libraries are customized according to the pathological characteristics of orthopedic subtypes, resulting in low screening efficiency and repetitive discoveries. Many studies still adopt isolated screening methods such as reporter gene assays or single phenotypic detection. The combinatorial strategy of virtual molecular docking coupled with *in vitro* screening and *in vivo* validation remains underdeveloped. Traditional HTS mostly employs endpoint detection, lacking real-time monitoring of cellular and molecular dynamics during the screening procedure. This may overlook transient regulatory effects and miss potential lead compounds.

For most candidates summarized in this review, current research remains confined to initial activity screening and preliminary validation. Critical gaps still exist: the direct molecular targets and upstream and downstream regulatory networks of most compounds are poorly defined. Few studies involve structural optimization, dose-effect analysis, or systematic pharmacokinetic evaluation. Natural products such as baicalein and daidzein are often limited by low bioavailability and metabolic instability. Repurposed drugs including phentolamine and panobinostat lack long-term safety evidence for orthopedic indications. Most screened bone repair materials are only validated for cell adhesion and osteogenic activity *in vitro*, with limited evaluation of *in vivo* mechanical stability, immune compatibility and long-term osseointegration. Furthermore, studies targeting the same signaling pathway rarely conduct horizontal comparisons in terms of pharmacological intensity, action sites and application scenarios, leaving research findings fragmented and difficult to form systematic theoretical guidance.

Another prominent limitation lies in multi-omics integration and data mining. Current HTS research is often decoupled from transcriptomic, proteomic and metabolomic datasets. Most screenings focus only on superficial phenotypic changes, without integrating multi-omics evidence to identify core regulatory genes and signaling hubs. Omics data heterogeneity and inadequate analytical algorithms hinder mechanistic interpretation at the molecular level. Furthermore, genetic background, age and gender variations are rarely considered in assay design, reducing the reproducibility and generalizability of screening results. The absence of unified evaluation and reporting standards also weakens comparability across different studies.

To address these limitations, future research can be directed toward several practical improvements. It is essential to develop more biomimetic screening models by combining organ-on-a-chip platforms with 4D bioprinting, to construct vascularized and innervated bone organoids integrated with mechanical stimulation and immune microenvironments. Custom compound and material libraries should be designed according to the pathological features of orthopedic diseases to improve screening specificity and efficiency. More emphasis should be placed on mechanistic exploration and translational validation, including target identification, signaling verification, structural modification and pharmacological assessment, to establish a complete research pipeline. Integrating artificial intelligence with multi-omics mining will facilitate the discovery of novel targets and clarify underlying molecular mechanisms. Unified experimental criteria and reporting norms should be established to enhance data comparability and facilitate secondary analysis. In addition, the development of low-cost HTS platforms will broaden its application in basic and grassroots research laboratories.

## Conclusion

In conclusion, HTS has become a powerful tool for drug and biomaterial discovery in orthopedic disorders. It has yielded abundant candidate molecules with considerable therapeutic potential. Nevertheless, current research is still constrained by insufficient biomimetic modeling, limited library diversity, superficial mechanistic exploration, and inadequate multi-omics integration. With further advances in organoid technology, artificial intelligence and microfluidics, HTS will evolve toward higher bionics, intelligence and clinical translatability. Continued optimization of screening frameworks will accelerate the discovery of novel therapeutic strategies, providing valuable references for precision treatment and bone regenerative repair in orthopedic clinical practice.

## Additional Information and Declarations

### Data Availability

The following information was supplied regarding data availability:

This is a literature review.
